# Implementation Status of Accrual Accounting System in Health Sector

**DOI:** 10.5539/gjhs.v7n1p24

**Published:** 2014-07-29

**Authors:** Mohammad Hossien Mehrolhassani, Akram Khayatzadeh-Mahani, Mozhgan Emami

**Affiliations:** 1 Assistant Professor, Research Center for Modeling in Health, Institute for Futures Studies in Health, Kerman University of Medical Sciences, Kerman, Iran; 2 Assistant Professor, Research Center for Health Services Management, Institute for Futures Studies in Health, Kerman University of Medical Sciences, Kerman, Iran; 3 Master of Health Services Management, Research Center for Health Services Management, Institute for Futures Studies in Health, Kerman University of Medical Sciences, Kerman, Iran

**Keywords:** health system reform, financial management, accrual accounting system

## Abstract

**Introduction:**

Management of financial resources in health systems is one of the major issues of concern for policy makers globally. As a sub-set of financial management, accounting system is of paramount importance. In this paper, which presents part of the results of a wider research project on transition process from a cash accounting system to an accrual accounting system, we look at the impact of components of change on implementation of the new system. Implementing changes is fraught with many obstacles and surveying these challenges will help policy makers to better overcome them.

**Methods:**

The study applied a quantitative manner in 2012 at Kerman University of Medical Science in Iran. For the evaluation, a teacher made valid questionnaire with Likert scale was used (Cranach’s alpha of 0.89) which included 7 change components in accounting system. The study population was 32 subordinate units of Kerman University of Medical Sciences and for data analysis, descriptive and inferential statistics and correlation coefficient in SPSS version 19 were used.

**Results:**

Level of effect of all components on the implementation was average downward (5.06±1.86), except for the component “management & leadership (3.46±2.25)” (undesirable from external evaluators’ viewpoint) and “technology (6.61±1.92) and work processes (6.35±2.19)” (middle to high from internal evaluators’ viewpoint).

**Conclusions:**

Results showed that the establishment of accrual accounting system faces infrastructural challenges, especially the components of leadership and management and followers. As such, developing effective measures to overcome implementation obstacles should target these components.

## 1. Introduction

Public organizations, all around the world, have employed managerial, organizational, and institutional change strategies so as to afford increased demand for financial accountability and more efficiency and effectiveness and transparency (Timoshenko, 2008). Generally, governments are implementing numerous business-like reforms which are recognized, in a broad level, as a new public management with the aim of aligning governmental sector with the private one (Harun, An, & Kahar, 2013). Among them, accounting reform is often the first step of public reforms and is considered the basic prerequisite for success of other public reforms (Eriotis, Stamatiadis, & Vasiliou, 2011).

Public financial accounting and reporting is a system which helps government’s accountability, improves financial decision-making and performance control by collecting, classifying, purifying and reporting financial information related to activities of public sectors (Bastani, Abolhalaj, Molania Jelodar, & Ramezanian, 2012). Over the last two decades, most developed countries have changed the basis of cash accounting applied in their public organizations in various accrual forms (Abolhalaj, Ramezanian, & Bastani, 2012) because in a cash accounting system, incomes are recognized and recorded at the time of receiving cash and expenses at the time of paying cash related to it (Monsen, 2001). Accordingly, the above system doesn’t provide information necessary for efficient and effective performance of the public (Peter, 2005). In accrual accounting system, on the other hand, economic events are recorded as soon as they occur. Thus, incomes are mainly recognized at the time of delivering services and selling goods, and expenses are recognized at the time of using assets and making commitments to generate income (Champoux, 2006). Therefore, a growing number of countries including New Zealand, Australia, Canada and America (Peter, 2005) have moved towards accrual accounting with the aim of possibility of evaluating managerial performances, ease in managing expenses, having access to comprehensive and useful information for allocating resources, financial reporting and better evaluation of management of cash accounts and assets (Abolhalaj et al., 2012).

In accounting literature over the past two decades, necessity of using complete accrual or balanced accrual bases has been taken into huge consideration in accounting governments’ non-business activities (Lande & Rocher, 2011). Most Accounting Standards Codification Boards in public sectors like Accounting Standards Boards of American state and local governments, Federal Government Accounting Advisory Board, Accountants’ International Federation, Accounting Standards Codification Board of public sector of Australian Accounting Research Foundation, Accounting and Auditing Committee of general sector of Elite Canadian Accountants’ Association and New Zealand Accountants’ Association have necessitated the application of complete and balanced accrual bases in accounting and financial reporting of governments’ non-business activities. International institutions like International Monetary Fund, Asian Development Bank and World Bank have also emphasized complete and balanced accrual bases (Karbasi Yazdi, & Tarighi, 2009).

In comparison with other public sectors, in health system of every country supplying and managing financial resources is one of the most essential and fundamental points which policy makers should consider. First steps to reform health financial management in Iran date back to 2003 during which a project was initiated (as a pilot) at 40 hospitals by Budget and Financial Resources Planning Office (BAFRPO) in Ministry of Health and Medical Education (MOHME) to evaluate cost price of services. To solve problems related to financial management of health sector, BAFRPO codified a project entitled “designing a new financial management system” (Hafez, Abolhalaj, & Ramezanian, 2009) in 4 phases. In the first phase, accrual accounting replaced cash accounting in all medical universities and medical faculties across the country and is now being implemented in all their affiliated units (Abolhalaj et al., 2012).

To obtain the given objective, it is necessary that the change be planned, designed and managed effectively and efficiently; in other words, organizational change strategies in different aspects of “structure, technology, duties and processes, manpower (decision-makers, executors and change supporters) and social, political, legal and economic variables” must be employed proportional to the occurred changes and status quo of the country (Rajput, Singh, & Singh, 2012). Employing organizational change theories and models including Kurt Lewin’s theory, Culture-Excellence model developed by Peters and Waterman and Kanter, and Processual approach by Pettigrew researchers developed a framework to help managers plan, monitor and evaluate changes andenable them to respond quickly to environmental fluctuations and to predict the changing patterns in the organization (Mehrolhassani & Emami, 2013). Undoubtedly, MOHME faces some challenges while implementing such changes; investigating these challenges, a better field can be provided to develop this system in health sector. As such, researchers tried to determine implementation challenges of the accrual accounting system in Kerman University of Medical Science (KUMS).

In his study, Bastani et al. examined financial management reform in Iran’s health sector in 2012; making use of in-depth and semi-structured interviews with financial managers of universities and members of strategic team of establishing new financial system as well as experts’ meetings. He concluded that low number of skilled and qualified manpower, people’s dissatisfaction due to high workload and low salary and job security, resistance to implementation and boredom at work, unawareness of the benefits of the new financial system by most senior managers of universities, complexity of software system and lack of organizational structure proportional to change were the most important implementations challenges of the accrual accounting system in Iran’s health sector (Bastani, Abolhalaj, Ramezanian, Jafari, & Rajab Kordi, 2012).

Windels and Christiaens carried out a study entitled “The Adoption of Accrual Accounting in Flemish Public Centers for Social Welfare” in 2007. In this study, an accounting index was used (in a big sample of Flemish local governments) to analyze adoption level of new accounting requirements. The study was based on organizational theory so as to establish a framework for analysis and interview to interpret information. In this study, not employing expert consultants and low interest of local managers were considered factors causing weak implementation of new accounting requirements (Windels & Christiaens, 2007).

Negash conducted a study entitled “Liberalization and the value relevance of accrual accounting information in South Africa”. In this study, panel data of 118 firms were used in a period of 17 years and the researcher concluded that in public sector, key factors such as nature of the objectives of public’s financial statements, assets, liabilities, equity, income and expenses, nature and role of financial statements have caused public activities to be interpreted differently from business organizations. In other words, making use of private accrual standards in public sector is not correct due to competitive constraints, heterogeneity of products and services. Thus, to employ positive points of accrual accounting system in public sector to improve decision-making system in managing resources, managerial reforms seem essential (Negash, 2008).

Using his experience as a financial manager of a health care system in Norway and interviewing process owners, Robbestad examined implementation of accrual accounting in his dissertation. He came to the conclusion that to change public accounting system, new organizational knowledge was required. Organizational knowledge will be achieved with the support of internal networks or outside the public sector. Moreover, changes in public rules and regulations, establishing feeling of people’s accountability and responsibility and supervising the way how works are done can help implement new system in entire sector. Hence, this change has been a step-by-step and gradual process (Robbestad, 2011).

## 2. Materials and Methods

The present research is an applied study carried out (using a quantitative method) in 2012 at KUMS.

Kerman is located in southeastern Iran and is, encompassing an area of more than 11 percent of Iran, considered one of the historical provinces of Iran. According to Statistical Center of Iran, Kerman had the population of 2,938,988 in 2011, with the metropolitan “Kerman” being its center. Kerman is the center of south east of Iran is an industrial, cultural, political, agricultural, academic-scientific and religious reference among other provinces in south east of Iran (The President’s Office Deputy of Strategic Planning and Control Statistical Center of Iran, 2011). Medical Science Universities are some of university centers of this province; of four universities located in Kerman, KUMS is considered the most important one and is the eighth largest medical sciences universities of Iran (Khayatzadeh-Mahani, Fotaki, & Harvey, 2013). About 7 towns with a population of 1,507,845 are covered by KUMS. It should be noted that in Iran’s health sector, educational system is integrated with service system (Khayatzadeh-Mahani et al., 2013; Baradaran, Shams-Hosseini, Noori-Hekmat, Tehrani-Banihashemi, & Khamseh, 2012).

Recently, some issues like ongoing expenses in health care system (Rkein, 2008) and ongoing expectations of customers in Iran’s health sector (Sevic, 2003) have caused Deputy of Resources Management and Planning Development of Ministry of Health to, in line with codifying and presenting new financial regulations for medical science universities of Iran, initiate a design entitled “new financial system” in 4 phases in a 5-year program. Thus, KUMS has taken necessary measures to change its financial system since early 2008 (Abolhalaj et al., 2012). Currently, 32 affiliated units of this university implement accrual accounting. Of them, only 13 units are dependent and the rest are independent. It is worth mentioning that only headquarter units and a few small cities such as research and technology deputy, food and drug deputy, student and cultural affairs deputy, educational deputy, health deputy, management and resources development deputy, treatment deputy, Rabor health center, Orsoiieh health center, Kuhbanan health center, Javadalaeme clinic, Besat clinic and emergency medical services are dependent units. Other cities’ hospitals affiliated with the KUMS and faculties have been declared as independent bodies. Demographic information and setting of the units are presented in Table 1.

**Table 1:** Demographic information of affiliated units of KUMS

Variables	Groups	Frequency	Frequency percentage
	Health networks and centers in towns of Kerman	52	22.7
	Hospitals and clinics affiliated to KUMS	91	39.7
	Emergency Medical Services	2	0.9
	Faculties affiliated to KUMS	18	7.9
Service area	Deputies of Kerman University of Medical Science	27	11.8
	Headquarters (University financial affairs management)	39	17.0
	
	Total	229	100

	High school diploma	75	32.8
	Associate degree	34	14.8
Education level	Bachelor’s degree	118	51.5
	Master’s degree	2	0.9
	
	Total	229	100

	Accounting	101	44.1
Academic field	Semi-relevant to accounting	46	20.1
	Irrelevant to accounting	82	35.8
	
	Total	229	100

Table 1 shows that most of the financial personnel work in hospitals and clinics affiliated to KUMS and few in emergency medical services. Concerning education level, Bachelor’s Degree and Master’s Degree had the highest and lowest frequency respectively. Moreover, academic field of most financial personnel of university was accounting and a few of them had studied in managerial and economic fields (semi-relevant to accounting).

In this study, we designed a questionnaire with Likert scale to evaluate the status of implementation of accrual accounting system at KUMS based on the preliminary framework resulted from a systematic review (Mehrolhassani, Abolhalaj, Nekoei Moghadam, Dehnavieh, & Emami, 2013), and in-depth and semi-structured interviews with 41 key experts in Iran’s health system on determining challenges of establishing accrual accounting system in Iran’s health sector. The questionnaire contained components such as management & leadership, followers, organizational structure, manpower, work processes, knowledge and technology. Designed questionnaire is divided into two parts. Part I included the general characteristics of the questionnaire Part II contained standards relating to performance of affiliated units of KUMS. The questionnaire incorporates three items related to leadership & management component, three items to follower’s component, six items to organizational structure component, seven items to manpower component, six items to work processes component, five items to knowledge component and four items to technology component. Validity of the questionnaire was confirmed according to researchers’ group meetings held to regulate the framework recognized in systematic review step (Mehrolhassani et al., 2013) and the preliminary report of interviews. Its reliability was measured using a pilot study and test-retest method and was confirmed according to correlation coefficient of 0.82, a significance level of 0.000 (p-value < 0.001) and Cranach’s alpha of 0.89. To gather evaluators’ views, 10 scores were allocated for every item written in the questionnaire (1-3: undesirable, 4-7: Moderate (4-5 average downward and 7-6: moderate to high and 8-10: desirable).

In order to make field observation and to evaluate performance, 32 units, as the study sample, were examined by internal evaluators (accrual system users) and external evaluators (university staff and researchers). Accordingly, 92 questionnaires were completed. To analyze data, descriptive (frequency), central (mean) and dispersion (standard deviation) parameters were used. Moreover, to compare all factors identified in independent and dependent units, T-Test was used. In addition, ANOVA test was used to compare health networks and centers, hospitals and clinics, emergency medical services, faculties and deputies according to different performance nature of units. Finally, to examine the relationship and correlation between the components, Pearson test was used. We employed SPSS software version 19 to analyze data and to perform statistical analysis.

## 3. Results

Table 2 shows the descriptive statistics of the studied variables. It is shown in this table that external evaluators optimistically scored all components higher than internal evaluators. Thus, the researchers divided average scores given to components into 2 groups of “average scores resulted from internal and external evaluators.” According to internal evaluators, the components “technology and followers” had respectively the highest and the lowest shares in implementing new accounting system at the given university. From external evaluators’ point of view, however, the component “management & leadership” played a lower role in implementing accrual accounting. It should be noted that both evaluations pointed to higher effect and intervention of technology in comparison with other components. Despite the differences found in evaluators’ scores, level of effects all components had on implementation of this accounting system was average downward in both evaluations, except for the component “management & leadership” (undesirable from external evaluators’ viewpoint) and “technology and work processes” (middle to high from internal evaluators’ viewpoint).

**Table 2:** Challenges identified in establishing accrual accounting system at KUMS

Items	Internal evaluation		External evaluation		Sum of both evaluations	

	Mean	Standard deviation	Mean	Standard deviation	Mean	Standard deviation
Management & leadership	5.23	2.56	3.46	2.25	4.34	2.40
Followers	4.94	2.56	4.15	2.10	4.54	2.33
Organizational structure	5.29	2.38	4.71	1.93	5	2.15
Manpower	4.98	2.26	4.51	1.99	4.74	2.12
Work processes	6.35	2.19	5.03	2.05	5.69	2.12
Knowledge	5.61	2.21	4.43	1.98	5.02	2.09
Technology	6.61	1.92	5.58	1.96	6.09	1.94

Total	5.57	2.29	4.55	2.03	5.06	1.86

Tables 3 and 5 also show the inferential statistics of the studied variables. According to normal distribution of variables among the studied sample, t-test and ANOVA tests were used to compare two groups of independent and non-independent units as well as all university affiliated units.

**Table 3:** Comparing components with normal distribution in 2 groups of independent and non-independent units

Components	Levene’s Test for Equality of Variances	Significance
Leadership & Management	0.8	✓ 0.50

		0.50

Followers	0.13	✓ 0.09

		0.08

Organizational Structure	0.48	✓ 0.13

		0.14

Manpower Component	0.78	✓ 0.005

		0.006

Work Processes	0.03	0.07

		✓ 0.09

Knowledge	0.45	✓ 0.04

		0.05

Technology	0.94	✓ 0.14

		0.13

**Independent Sample T-Test**		

**Table 4:** Status of components with normal distribution in 2 groups of independent and non-independent units

Components	Nature of units	Sum of both evaluations	

		Mean	Standard deviation
Leadership & Management	Independent	4	2.21

	Dependent	3.67	2.22

Followers	Independent	5.33	2.17

	Dependent	4.50	1.75

Organizational Structure	Independent	5.33	1.68

	Dependent	4.33	1.42

Manpower Component	Independent	5	1.50

	Dependent	4.28	1.53

Work Processes	Independent	6.17	1.50

	Dependent	5.86	2.05

Knowledge	Independent	5	1.50

	Dependent	4.90	1.70

Technology	Independent	6.25	1.45

	Dependent	5.75	1.42

**Table 5:** Comparing components with normal distribution in all affiliated units of KUMS

*	Management & leadership	followers	Organizational structure	Manpower	Work processes	Knowledge	Technology
significance	0.44	0.06	0.16	0.52	0.33	0.84	0.29

**One-Way ANOVA Test**							

Table 3 shows that the components “manpower and knowledge including knowledge of members of change team in the field of managing and implementing accrual accounting system” were different in both groups of independent and dependent units and that the relationships and probabilities observed here weren’t by chance. Also, differences found between status of followers and work processes were moderate (0.0) and it was difficult to judge in this regard (because 0.1 < p-value < 0.05).

In all components, independent units had better performance and status regarding implementation of accrual system and were involved more in implementation (Table 4).

Table 5 shows that the difference between status of all components was not significant in all units and that the possibility of presence of a difference among health centers and networks, hospitals and clinics, emergency medical services, faculties and deputies was low (because p-value > 0.05), except for the component “followers” which was moderate (because 0.1 < p-value < 0.05).

Table 6 reflects the results obtained from examining the relationship and correlation between the studied components. Results show that there is a direct and significant relationship between all components (p-value<0.05). The following results show the highest relationship between components with correlation coefficient of more than 0.6:

**Table 6:** Correlation coefficient among studied components

*		Management & leadership	Followers	Organizational structure	Manpower	Work processes	Knowledge	Technology
Management & leadership	Pearson correlation coefficient	1	0.66	0.66	0.67	0.52	0.73	0.40

	significance	-	0.000	0.000	0.000	0.000	0.000	0.000

Followers	Pearson correlation coefficient	0.66	1	0.82	0.73	0.52	0.70	0.44

	significance	0.000	0.000	0.000	0.000	0.000	0.000	0.000

Organizational structure	Pearson correlation coefficient	0.66	0.82	1	0.82	0.51	0.68	0.54

	significance	0.000	0.000	-	0.000	0.000	0.000	0.000

Manpower	Pearson correlation coefficient	0.66	0.73	0.82	1	0.68	0.79	0.53

	significance	0.000	0.000	0.000	-	0.000	0.000	0.000

Work processes	Pearson correlation coefficient	0.52	0.52	0.51	0.68	1	0.65	0.52

	significance	0.000	0.000	0.000	0.000	-	0.000	0.000

Knowledge	Pearson correlation coefficient	0.73	0.70	0.68	0.79	0.65	1	0.52

	significance	0.000	0.000	0.000	0.000	0.000	-	0.000

Technology	Pearson correlation coefficient	0.40	0.44	0.54	0.53	0.52	0.52	1

	significance	0.000	0.000	0.000	0.000	0.000	0.000	-

1) Component “knowledge” with components “organizational structure, work processes, management & leadership, followers and manpower”;
2) Component “organizational structure” with components “knowledge, management & leadership, followers and manpower”;
3) Component “work processes” with components “knowledge and manpower”;
4) Component “management & leadership” with components “followers and manpower”;
5) Component “followers” with component “manpower”.

## 4. Discussion and Conclusions

According to challenges identified in interviews and the systematic review framework (Mehrolhassani et al., 2013), 8 components affecting the implementation of new accounting system were identified and evaluated in KUMS. These components include management & leadership, followers, organizational structure, manpower, work processes, knowledge and technology. Field observation of all of the above-mentioned components showed that all components had middle to low status except for technology which had middle to high status.

Results suggested that the status of management & leadership was middle downward and had the lowest status in comparison with all other components. In fact, managers and bosses of the university affiliated units had lower interest, awareness and commitment towards implementation of the accrual accounting system in their units and had no cooperation in obtaining accrual reports of the system and no support for the change implementation.

This result is consistent with the results obtained by Babakhani (2006) and Windels and Christiaens (2007); Babakhani proposed that moving towards accrual accounting must be based on a gradual change approach; he also stated that causing a fundamental change in Iran’s financial accounting and reporting system may meet the required general conditions and may require fundamental changes in authorities’ attitudes towards accountability culture and in their firm and true beliefs in accountability to citizens, i.e. respond-seekers and real owners of justice (Babakhani, 2006).

The component “followers” had average downward status in all affiliated units. It was clarified that the followers hadn’t been formed in these units as an implementation driving force and that their members weren’t involved in cooperation and interaction necessary for establishing the system. In his research carried out with the aim of identifying factors affecting the adoption of the accrual accounting system in public sector, Christensen showed that sufficient guidelines and supportive activities must be presented by skilled experts like managerial consultants and that predicted objectives of this huge change will fade away without external control and auditing (Christensen, 2002).

Status of organization structure was middle to low in implementing accrual accounting, i.e. there existed no organizational chart proportional to changes in the affiliated units, and accountability lines and integration of decision-making authority were weakened due to lack of cooperation of the unit bosses. These issues, in turn, affected organizational relationships. In his study, Bastani et al. stated that absence of an organizational structure proportional to changes and absence of a suitable performance evaluation system were the most important structural problems of the change (Bastani et al., 2012).

Manpower status was also average downward in evaluating performance of the units. As shown in Table 2, academic field of only 44.1% of financial personnel was accounting. Hence, every unit faced shortage of skilled and qualified manpower due to personnel’s insufficient skill and experience, lack of training proportional to users’ needs and involvement of people with different organizational posts and irrelevant academic certificates. Results of a study carried out in 2003 with the aim of examining dimensions of responsibility and role of accounting in encountering accountability requirements in Islamic Republic of Iran are consistent with the results of this study. Mahdavi and Funnel reached a conclusion that manpower was considered the first member in changing accounting system of public sector. Shortage of expertise and competence, lack of clear understanding of the process and obstacles in accrual accounting have resulted in not enjoying the expected benefits of the new system. Thus, government must provide accountants of public sectors with educational opportunities; this kind of education must also be considered for managers who use accrual information (Mahdavi & Funnel, 2003).

Work processes are in fact steps through which system input (i.e. financial events, people’s knowledge and skill, software and hardware facilities, etc.) changes to system output (that is, preparing clear reports and financial statements of an accrual system). These steps can, for example, be considered the way people interact with one another at work, the way accrual software is used and the way accounts are recorded equally in the system, etc. However, result showed that full details of working with software, recording all operations equally in all affiliated units and sending financial information to accrual user by financial units were not clear and standard. Results of the study by Bastani et al. also pointed to lack of a strategy, an operational plan and feedback and project outputs in implementing accrual accounting system in Iran’s health sector (Bastani et al., 2012).

Bosses’ knowledge of change management, competence of members of change team and experience and skill of owners of implementation process at KUMS was average. These results matched the results of the studies by Wynne (2004), Negash (2008) and Windels and Christiaens (2007). It was clarified in his research that introducing accrual accounting in public sectors was a costly and time-consuming process which required cooperation of a wide range of key beneficiaries, fundamental changes in organization, processes, procedures and accountability of managers, presence of technical knowledge in users (Negash, 2008), employing abilities of specialized consultants in this field (Windels & Christiaens, 2007) and a suitable auditing system able to control users’ performance (Wynne, 2004).

Unlike other components, technology (scored average to high) was the most appropriate one for implementing accrual accounting at KUMS in terms of software and hardware infrastructures, suitability and people’s access to the described processes. However, it is still not complete. It was mentioned in a report in 2005 in London that governments must prepare and present content and form of their reports proportional to their users’ needs (Treasury Staff Great Britain, 2005).

Overall, the status of all components affecting the establishment of accrual accounting system in KUMS faces infrastructural challenges, especially the components of leadership and management and followers. As such, developing effective measures to overcome implementation obstacles should target these three components.

## 5. Recommendations

Generally, the following steps are suggested for improving the establishment of new accounting system in public sectors:

1) Codifying a change plan in every organization requires, in the first place, commitment and support of senior managers who are responsible for planning and supporting it.
2) Accrual system administrators must identify problems and obstacles in the change process. Recognizing problems requires a general analysis of threats, opportunities and weak and strong points that the organization is faced with.
3) In the next step, an appropriate timing must be made so as to implement necessary changes proportional to the change. The first vital measure in this step is to change organizational culture which, in fact, is considered a basis for changing individuals’ behaviors and their decisions. Changes like improving the ability of unit managers in the field of change management, training them how to use accrual information, justifying them about applying accrual information in their organizational decision-makings, forming change teams, improving their abilities in implementation aspect, educating, justifying, motivating and preparing accrual users for changes, codifying strategies and operational plans and establishing a suitable reward system should not be underestimated in this step.
4) After making sure of performing all of the above-mentioned steps, change can be implemented. However, evaluation is essential to implement the system successfully because evaluating the programs is necessary to monitor progress, identify barriers and determine factors affecting success or failure of future programming.

## 6. Limitations of the Study

Owing to the large area covered by the KUMS, limited facilities of evaluators and non-cooperation of evaluators in filling questionnaires of some units, evaluation of all units was difficult. Moreover, evaluators’ pessimistic and optimistic viewpoints were hard to identify.
